# Superconductivity below 20 K in heavily electron-doped surface layer of FeSe bulk crystal

**DOI:** 10.1038/ncomms11116

**Published:** 2016-04-06

**Authors:** J. J. Seo, B. Y. Kim, B. S. Kim, J. K. Jeong, J. M. Ok, Jun Sung Kim, J. D. Denlinger, S. -K. Mo, C. Kim, Y. K. Kim

**Affiliations:** 1 Institute of Physics and Applied Physics, Yonsei University, Seoul 120-749, Korea; 2 Center for Correlated Electron Systems, Institute for Basic Science, Seoul 151-742, South Korea; 3 Department of Physics, Pohang University of Science and Technology, Pohang 790-784, Korea; 4 Advanced Light Source, Lawrence Berkeley National Laboratory, Berkeley, California 94720, USA; 5 Department of Physics and Astronomy, Seoul National University, Seoul 151-747, Korea

## Abstract

A superconducting transition temperature (*T*_c_) as high as 100 K was recently discovered in one monolayer FeSe grown on SrTiO_3_. The discovery ignited efforts to identify the mechanism for the markedly enhanced *T*_c_ from its bulk value of 8 K. There are two main views about the origin of the *T*_c_ enhancement: interfacial effects and/or excess electrons with strong electron correlation. Here, we report the observation of superconductivity below 20 K in surface electron-doped bulk FeSe. The doped surface layer possesses all the key spectroscopic aspects of the monolayer FeSe on SrTiO_3_. Without interfacial effects, the surface layer state has a moderate *T*_c_ of 20 K with a smaller gap opening of 4.2 meV. Our results show that excess electrons with strong correlation cannot induce the maximum *T*_c_, which in turn reveals the need for interfacial effects to achieve the highest *T*_c_ in one monolayer FeSe on SrTiO_3_.

## Abstract (web summary)

Thin FeSe film on SrTiO_3_ substrate becomes a superconductor with a transition temperature over 100 K, yet the origin remains controversial. Here, Seo *et al*. show superconductivity below 20 K on the electron-doped surface of an FeSe crystal, suggesting a decisive role of interfacial effects in the enhancement of superconductivity.

## Graphical abstract

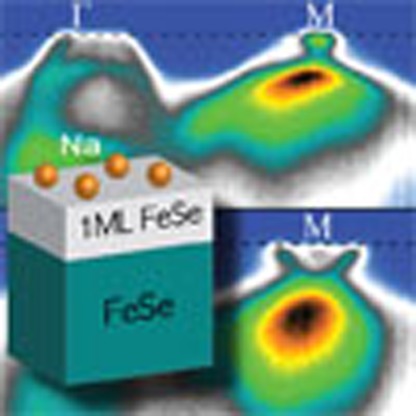

A strikingly enhanced superconducting transition temperature (*T*_c_), far above the previous record of *T*_c_ in bulk iron-based superconductors, was discovered in a relatively simple system of one monolayer (1 ML) FeSe on SrTiO_3_ (STO)^1^,^2^,^3^,^4^,^5^,^6^. The observation quickly initiated extensive and intensive studies to unveil the key mechanism for the enhancement. The mechanism, if found, should be important in its own right, but may also provide key information on the superconducting mechanism in iron-based superconductors.

Two views are mainly considered on the issue at present. In the first view, the origin of the enhancement comes from the FeSe layer. Angle-resolved photoemission spectroscopy (ARPES) studies have shown that 1 ML FeSe on STO is heavily electron-doped with electrons provided by the substrate and, as a result, has only electron pockets^7^,^8^. The observed electron bands are also found to have insulator–superconductor crossover with an enhanced electron correlation strength that is possibly due to confinement of electrons in two-dimensional (2D) state or strain from the substrate^9^,^10^. It was then proposed that 1 ML FeSe/STO shares the same superconducting mechanism with ordinary iron-based superconductors that are considered to be strongly correlated electron systems as cuprates^11^,^12^,^13^.

In the other view, the origin comes from outside of the FeSe layer. That is, a strong interfacial effect is an essential ingredient of the large *T*_c_ enhancement^8^,^14^,^15^,^16^,^17^. This view is based on the fact that the enhanced *T*_c_ is observed only near the interface^8^,^17^. As for what exactly the interface effect is, two possibilities have been raised so far. The first one is an additional pairing channel provided by the STO phonons^15^. The observation of a replica band, believed to be a fingerprint of strong coupling between an electron in the FeSe layer and an optical phonon mode of the underlying STO, suggests a significant role of such additional pairing channel^15^. The other possibility comes from the stabilization of an ordered state by the interface that should provide strong spin fluctuation when it is broken by electron doping. This view is based on an earlier experimental observation that the phase transition temperature increases with less number of layers^8^.

So far, there is no experimental result that can clearly reveal the dominant mechanism. A simple way to address the issue would be to fabricate a free standing 1 ML FeSe with excess electrons. It can clearly tell us if the interface effect is needed to achieve the enhanced superconductivity, but is practically impossible to achieve. Instead, our idea is to closely mimic the situation by inducing a monolayer-like FeSe state on a FeSe bulk crystal via surface electron doping that can be done by alkali metal evaporation^18^,^19^. In the electronic structure point of view, the induced state is found to possess all the key characteristic aspects of 1 ML FeSe/STO: heavy electron doping, reduced dimensionality (2D) and enhanced electron correlation strength. It is thus almost identical to 1 ML FeSe/STO, with the only difference being the lack of the interface effect. Therefore, the resulting *T*_c_ in the induced state should tell us what the main ingredients for the enhanced *T*_c_ in 1 ML FeSe/STO are. We now demonstrate that the induced state on bulk FeSe indeed satisfies these three characteristics of 1 ML FeSe.

## Results

### Electronic structures and surface electron doping

**Figure 1:**
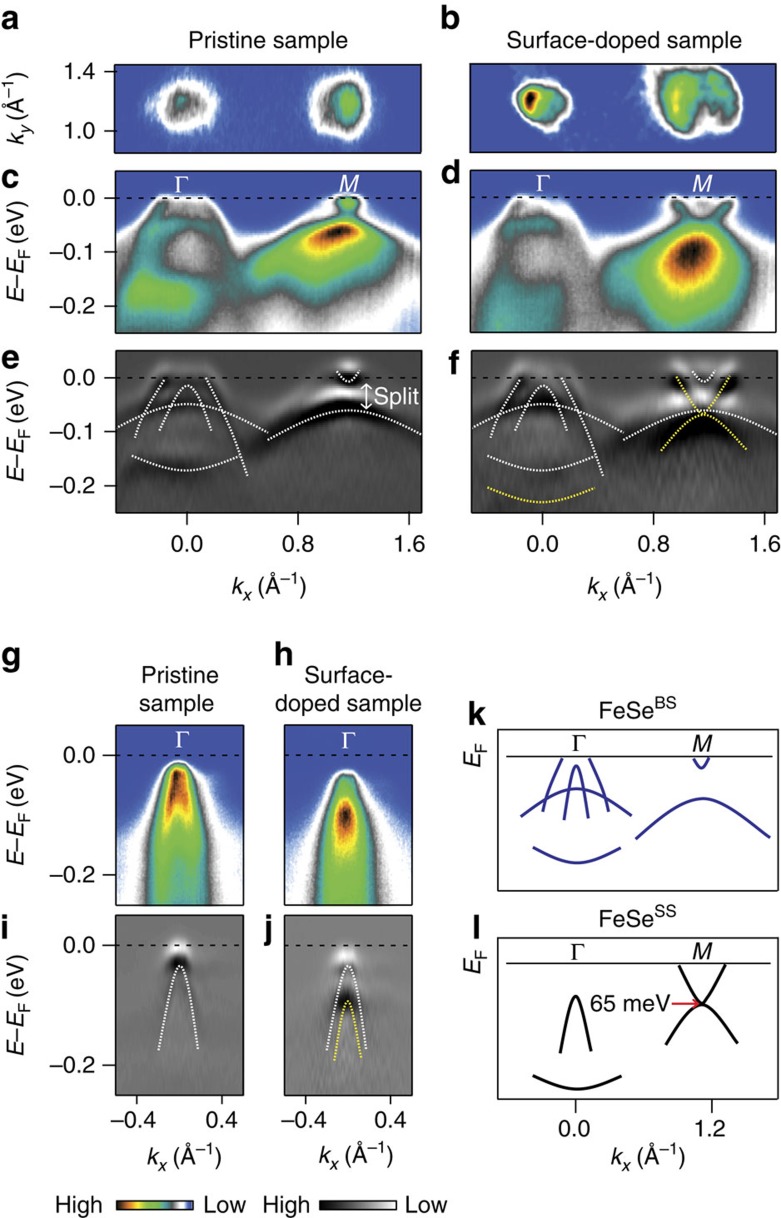
Electronic structures of pristine and surface electron-doped FeSe. (**a**) Fermi surface mapping of pristine and (**b**) surface-doped FeSe, measured at 30 K. (**c**) Band dispersions along the Γ-*M* high-symmetry line of pristine and (**d**) surface-doped FeSe, and (**e**,**f**) second derivatives of **c** and **d**. White and yellow dashed lines indicate the band dispersions of pristine and surface-doped FeSe, respectively. (**g**) Band dispersion around the Γ-point in a different geometry for pristine and (**h**) surface-doped FeSe. (**i**,**j**) Second derivatives of **g** and **h**. Schematics for the band dispersions of the (**k**) bulk state (FeSe^BS^) and (**l**) doping induced surface state (FeSe^SS^).

We first show that the doping level achieved via surface electron doping can reach that of the 1 ML FeSe/STO. Figure 1c,d shows the band dispersions along the Γ–*M* high-symmetry line of pristine and surface-doped samples measured at 30 K. The electron band in surface-doped sample has a downward shift with a larger Fermi surface (Fig. 1a,b). The observed shift, judging from the electron band bottom location at 65 meV, is similar to the value for 1 ML FeSe/STO^4^. The doping level estimated from the Fermi surface volume is 0.1 electrons per Fe, a value similar to that of 1 ML FeSe/STO with the *T*_c_ of 55–65 K (0.1–0.12 electrons per Fe)^4^,^7^. As for the hole band, it first looks as if there is not much change in the dispersion upon surface doping. However, a close inspection of the data taken with various geometries shows a downward shift of the hole band (Fig. 1g,h). In addition, a tiny and faint electron band at the *M*-point still remains with the size very close to that of the pristine sample. The observation of both surface and bulk states can be understood to be from different length scales of the charge doping and probing depth. That is, the probing depth of ARPES is larger than the charge doping depth and, as a consequence, signals from both the doped surface and underlying bulk states are seen. From now on, we denote the bulk state as FeSe^BS^ and doping induced surface state as FeSe^SS^.

A notable aspect of the band dispersion in FeSe^SS^ is that it does not have the split bands near the *M*-point that are believed to be a manifestation of the ferro-orbital ordering^20^,^21^,^22^. This suggests that ferro-orbital ordering is suppressed through the surface electron doping. With both the hole and electron bands simply shifted to the higher-binding energy side and the ferro-orbital ordering suppressed, the overall band dispersion of FeSe^SS^ fully replicates that of the 1 ML FeSe/STO. The full band assignments are made with the second derivative data in Fig. 1d,f,i,j, and the results are summarized in Fig. 1k,l for FeSe^BS^ and FeSe^SS^, respectively.

### Reduced dimensionality and enhanced correlation

**Figure 2:**
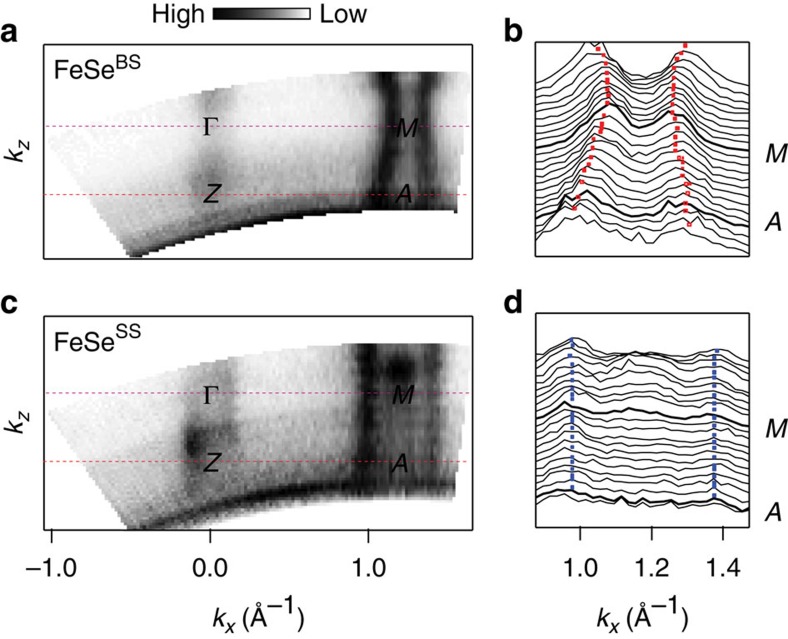
*k*_*z*_ dependences of the bulk state and doping induced surface state. (**a**) Constant energy map in the *k*_*x*_–*k*_*z*_ plane from bulk state (FeSe^BS^) normal state at 120 K and (**b**) stacked momentum distribution curves (MDCs) for the electron band. The markers denote local maximum points and represent the band position. (**c**) *k*_*z*_ dependence for surface state (FeSe^SS^) at 30 K and (**d**) stacked MDCs for the electron band.

We next show that the doping induced state on the surface is almost 2D. If the state is 2D, there is no out-of-plane momentum (*k*_*z*_) dependence (or photon energy dependence in the experiment) in the band structure. Figure 2a,c shows Fermi surface maps of pristine and surface-doped sample in the *k*_*z*_–*k*_*x*_ plane. The out-of-plane dispersion data for FeSe^BS^ was taken at 120 K to avoid complications from the ordered phase, while FeSe^SS^ data was taken at a lower temperature of 30 K. Figure 2b,d shows stacked momentum distribution curves near the *M*-point. FeSe^BS^ has a weak but clear three-dimensional electronic structure modulation in both hole and electron Fermi surfaces. On the other hand, the FeSe^SS^ case given in Fig. 2c,d shows no modulation along the *k*_*z*_ direction. This implies that the state is confined within a 2D layer or, at least, it has negligibly weak inter-layer interaction. We conclude the former is the case, as will be discussed later. In fact, we believe that only the very first layer of FeSe is doped.

**Figure 3:**
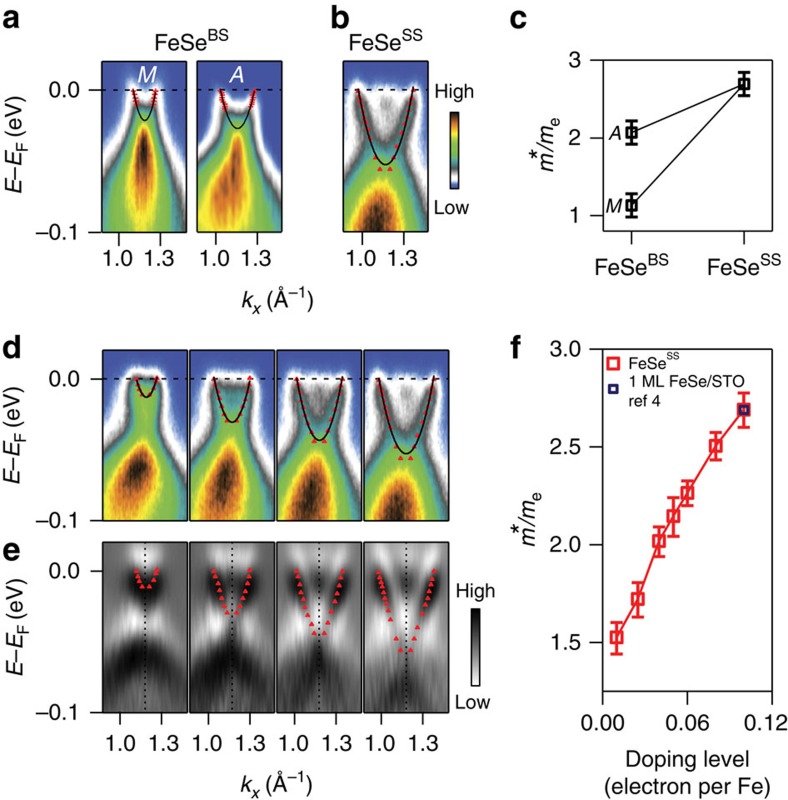
Evolution of the effective mass in FeSe^SS^. (**a**) Electron band near the *M*-point at *k*_*z*_=0 (*π*) before surface electron doping. Overlaid on each plot is a parabolic fit used in extracting the effective mass. (**b**) The same cut after surface electron doping. (**c**) Effective masses of the FeSe^BS^ and FeSe^SS^ at different *k*_*z*_. (**d**) Evolution of the electron band with surface electron doping. Parabolic fits of the peak positions (red triangles) are overlaid. (**e**) Second derivatives of **d**. Red triangles are peak positions. (**f**) Evolution of the effective mass from FeSe^BS^ to FeSe^SS^ as a function of surface-doping level. The error bars represent the s.d. of the parabolic fitting.

Another characteristic feature of the 2D confinement is the strengthening of the electron correlation^10^,^23^,^24^,^25^,^26^,^27^. A way to examine the electron correlation strength is to check the effective mass. The effective mass can be obtained from a parabolic fit of the experimental band dispersion in Fig. 3a,b. As shown in Fig. 3c, the effective mass of FeSe^SS^ state is m*/*m*_e_=2.7 (*m*_e_ is the free electron mass), larger than that of FeSe^BS^ (m*/*m*_e_=1.1 and 2.1 at *k*_*z*_=0 and *π*, respectively). It clearly indicates a stronger electron correlation in FeSe^SS^. We also note that the evolution of the effective mass upon surface electron doping in Fig. 3d–f shows a gradual increase without any abrupt change. It strongly suggests that no phase transition is involved in the observed change in the effective mass.

### Confinement of doped electrons within the first layer

**Figure 4:**
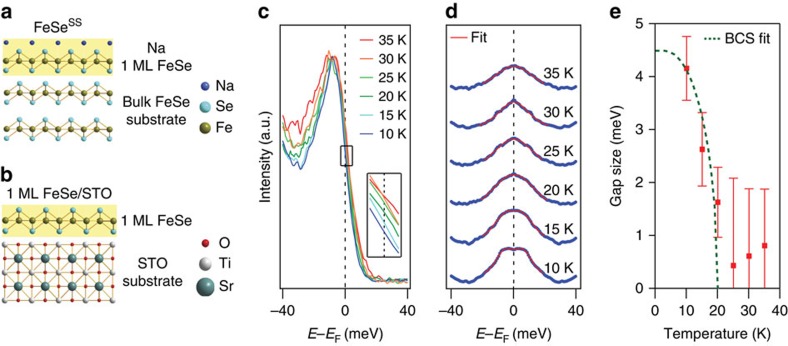
Superconducting gap of the FeSe^SS^ state. (**a**) Schematic views of the doping induced surface state FeSe^SS^ and (**b**) 1 ML FeSe/STO. (**c**) Temperature-dependent EDCs taken at the Fermi momentum of the electron pocket at the *M*-point. (**d**) Symmetrized EDCs of **c**. Each spectrum is fitted with a Dynes function and the result is overlaid as a solid red curve. (**e**) Temperature-dependent superconducting gap size extracted from the fit. The dashed green line is the Bardeen, Cooper and Schrieffer (BCS) gap function. The error bar was determined by the combination of experimental resolution and the s.d. of the fitting process.

As mentioned above, we believe the doping is confined only within the very first layer, possibly due to the weak van der Waals coupling between FeSe layers. The first evidence is its large effective mass and absence of the *k*_*z*_ dispersion. It was recently reported that the band width of the electron pocket at the *M*-point of 1 ML FeSe/STO is insensitive to the lattice constant of the substrate^28^. This implies that the effective mass of the electron pocket is hardly affected by strain and is thus almost solely determined by the dimensionality of the system. Therefore, the effective mass almost the same as that of 1 ML FeSe/STO tells us that FeSe^SS^ is likely confined within a layer as is the case for 1 ML FeSe/STO. Another evidence for a single-layer doping is that no multilayer band splitting is observed. The sharp and clear band features in the spectra in Fig. 1d also supports our view. If electron permeates into several layers with a potential gradient, then the band should be broad since we will then measure the sum of bands with different dopings. With all these evidences, we conclude that the doped electrons reside almost within the first layer of FeSe. FeSe^SS^ thus can be regarded as 1 ML FeSe on bulk FeSe without interface effects such as strain (illustrated in Fig. 4a,b).

### Superconducting gap

As we have demonstrated that the induced state is almost identical to 1 ML FeSe/STO in the electronic structure point of view, the next step is to check how the *T*_c_ changes in the induced state. Figure 4 shows the result of superconducting gap measurements. Leading edge shift upon cooling is captured in the raw energy distribution curves (EDCs) from the Fermi momentum of the electron band (Fig. 4c). Symmetrized EDCs given in Fig. 4d show a gap feature at the lowest temperature with a size of 4.2 meV(± 0.6), obtained by fitting the data with a Dynes function^29^. Temperature dependence of the gap size in Fig. 4e roughly traces the mean field order parameter dependence with a *T*_c_ of 20 K, which results in 2Δ/*k*_B_*T*_c_ of about 5, where *k*_B_ is the Boltzmann constant. This is somewhat smaller than but not too different from 6–7 for 1 ML FeSe/STO^7^.

## Discussion

Summarizing the experimental results, judging from observation of the 2D dispersion of the surface bands and absence of multilayer band splitting, we find only the very top surface layer of FeSe is doped upon Na evaporation. The doping level (0.1 electrons per Fe) and effective mass (2.7 *m*_e_) are identical or very similar to those of 1 ML FeSe/STO. As a result, FeSe^SS^ has heavy electron doping, 2D confined state within a layer and strong correlation, that is, all the key characteristics of 1 ML FeSe/STO in the electronic structure.

The increase in the effective mass in FeSe^SS^, about twice larger than that in FeSe^BS^, is from the 2D confinement of the doped electrons. A higher effective mass means more states available for the superconductivity near the Fermi energy. It should be pointed out that, while the increase in the effective mass is expected from reduced dimensionality in general (due to reduction in hopping channels), it should be mostly from enhanced electron correlation because FeSe^BS^ is already almost 2D as seen in Fig. 2a. We note that there are reports suggesting that enhanced correlation may enhance the *T*_c_^9^,^10^. These results suggest that increased effective mass and strong electron correlation resulting from electron doping can enhance *T*_c_.

On the other hand, gap analysis of our data shows that FeSe^SS^ has a *T*_c_ of ∼20 K, a value much lower than that of 1 ML FeSe/STO. It was very recently reported that surface-doped 30 ML FeSe film with K evaporation has a *T*_c_ of ∼40 K (ref. 30). We believe that only the top layer of their thin film was also doped, considering our findings. In addition, Li_0.84_Fe_0.16_OHFe_0.98_Se was reported to have 2D electronic structure with a *T*_c_ of 41 K because of the enlarged spacing between FeSe layers from (LiFe)OH intercalation^31^. Even though it is still to be understood why the *T*_c_ of our surface-doped bulk FeSe is lower than other systems, all the results including ours point to the notion that electron doping with strong correlation can increase the *T*_c_ only to a limited value of ∼40 K.

The lower *T*_c_ found in FeSe^SS^ should be attributed to its difference from 1 ML FeSe/STO. A clear distinction of FeSe^SS^ from 1 ML FeSe/STO is the absence of the interface effects. It therefore suggests that the highest *T*_c_ observed in 1 ML FeSe/STO requires interface effect in addition to the heavy electron doping and strong correlation. The exact role of interface effect is not clear yet. As mentioned earlier, the interface effect could be a direct contribution from interface phonons such as STO optical phonons^15^, or an indirect one through strengthening of the ferro-orbital ordering in undoped and subsequent enhancement of the associated fluctuation upon doping^8^. Even though it is yet to be seen which of the two is responsible and further studies are needed to resolve the issue, it is clear that the interface effects are needed to achieve the high *T*_c_ in 1 ML FeSe/STO.

## Methods

### ARPES measurement

ARPES measurements were performed at the Beamline (BL)10.0.1 and 4.0.3 of the Advanced Light Source. Surface electron doping was done by Na evaporation on the sample surface using commercial SAES alkali metal dispensers. Spectra were taken with Scienta R4000 (BL 10.0.1) and R8000 (BL 4.0.3) electron analysers with overall energy resolutions of 10 meV (BL 10.0.1) and 13 meV (BL 4.0.3), respectively. Photon energy-dependent measurements were performed with photon energies from 50 to 90 eV. The samples were cleaved and doped at 30 K in an ultrahigh vacuum better than 4 × 10^−11^ torr. All measurements were performed within 1 h per sample because of the short surface life time after Na evaporation.

### Data analysis

The Fermi surface volume of the electron pocket was estimated by assuming two elliptical Fermi surfaces perpendicular to each other with *k*_F_: *a*=0.46 Å^−1^ and *b*=0.38 Å^−1^ extracted from high-symmetry line band dispersions. The symmetrized spectra in Fig. 4d were obtained by folding the spectra with respect to *E*_F_ after dividing them by the corresponding Fermi-Dirac distribution function. For superconducting gap analysis, Dynes function given in ref. 29 was used. Temperature dependence of the gap was fitted with the Bardeen, Cooper and Schrieffer functional form in the weak-coupling limit as given by *E*_g_(T)=*E*_g_(0) tanh (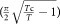) (refs 32, 33).

## Additional information

**How to cite this article**: Seo, J. J. *et al*. Superconductivity below 20 K in heavily electron-doped surface layer of FeSe bulk crystal. *Nat. Commun.* 7:11116 doi: 10.1038/ncomms11116 (2016).
